# A network analysis of executive deficits in patients with psychosis and their healthy siblings

**DOI:** 10.1192/j.eurpsy.2021.1388

**Published:** 2021-08-13

**Authors:** G. Gil-Berrozpe, A. Sánchez-Torres, R. Lorente-Omeñaca, L. Moreno-Izco, E. García De Jalón, R. Hernandez Anton, V. Peralta, M. Cuesta

**Affiliations:** 1 Mental Health Group, Instituto de Investigación Sanitaria de Navarra (IdISNA), Pamplona, Spain; 2 PsiquiatrÍa, COMPLEJO HOSPITALARIO DE NAVARRA, PAMPLONA, Spain

**Keywords:** Executive functions, WCST, Network analysis, Exploratory graph analysis

## Abstract

**Introduction:**

Psychopathological symptoms and cognitive impairment are core features of patients with psychotic disorders. Executive dysfunctions are within the most commonly observed deficits and the Wisconsin Card Sorting Test (WCST) is the test most extensively used for their assessment. Yet, the structure of executive deficits remains unclear, as there may be different underlying processes.

**Objectives:**

The study’s aims were to explore and compare the network structure of the WCST measures in psychosis and their unaffected siblings.

**Methods:**

Subjects were 298 patients with a DSM 5 diagnosis of psychotic disorder and 89 of their healthy siblings. The dimensionality and network structure of the 13 WCST measures were examined by means of the Exploratory Graph Analysis (EGA) and centrality parameters.

**Results:**

The WCST network structure comprised 4 dimensions: Perseveration (PER), Inefficient sorting (IS), Failure to maintain set (FMS) and Learning (LNG). Patient and sibling groups showed a similar network structure and in both cases the network structure was reliably estimated.

**Figure:**
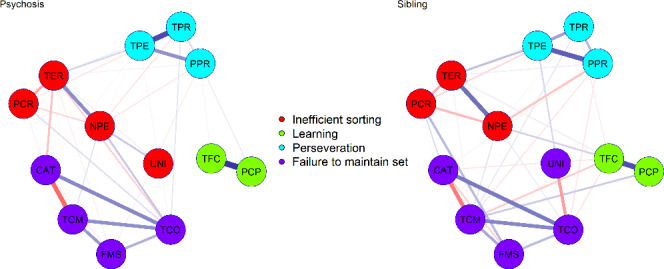

**Figure:**
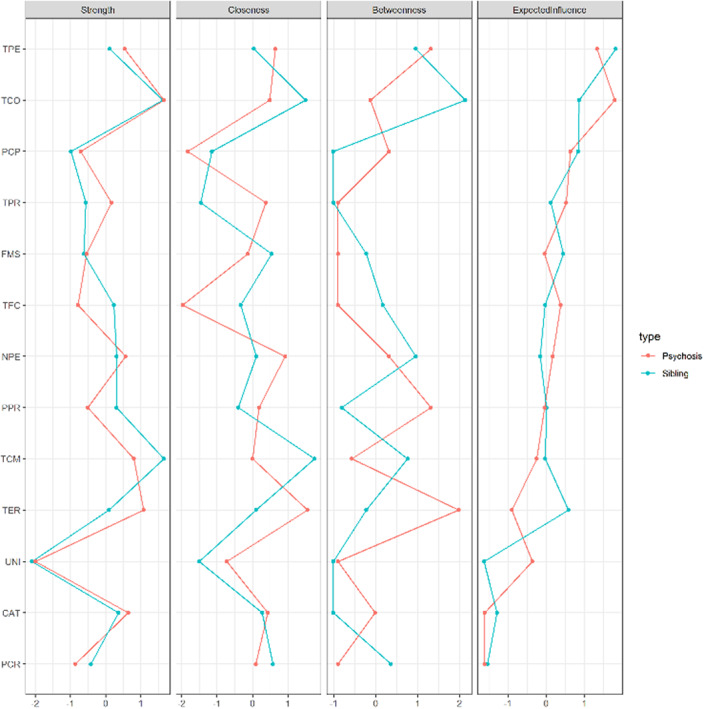

**Conclusions:**

Perseveration measures reflect the inability to switch sorting rules when necessary. Scores for the IS dimension can occur when the subject ineffectively tries to test different sorting hypotheses, changing at random the response. FMS reflects the subject’s strategy when he/she is able to find out the sorting rule, but is unable to keep applying that rule long enough. LNG comprised conceptual ability and learning items. The lack of significant difference between network structures is in keeping with results from exploratory and confirmatory studies demonstrating an invariant cognitive factor structure between schizophrenia patients and their unaffected siblings.

